# Biology and phenology of three leaf beetle species (Chrysomelidae) in a montane forest in southeast Brazil*

**DOI:** 10.3897/zookeys.547.9015

**Published:** 2015-12-17

**Authors:** Vivian Flinte, Ethel Hentz, Barbara Mascarenhas Morgado, Anne Caruliny do Monte Lima, Gabriel Khattar, Ricardo Ferreira Monteiro, Margarete Valverde de Macedo

**Affiliations:** 1 Av. Carlos Chagas Filho, 373. CCS, IB, Laboratório de Ecologia de Insetos, Sala A0-111, Universidade Federal do Rio de Janeiro, Ilha do Fundão, CEP 21941-590, CP 68020, Rio de Janeiro, RJ, Brasil

**Keywords:** Population fluctuation, viviparity, host plant, altitude, climate

## Abstract

The population phenology of the cassidines, *Coptocycla arcuata* and *Omaspides trichroa*, and the chrysomeline, *Platyphora axillaris*, was studied at Serra dos Órgãos National Park, State of Rio de Janeiro, southeast Brazil. Monthly surveys of larvae and adults were conducted between 2008 and 2011 at approximately 1000 m altitude on their respective host plants, *Cordia polycephala* (Boraginaceae), *Ipomoea philomega* (Convolvulaceae) and *Solanum scuticum* (Solanaceae). This is the first observation of larviparity and host record for *Platyphora axillaris*. Although having different life history traits, all species showed similar phenologies. They were abundant from October to March, months of high temperatures and intense rainfall, with two distinct reproductive peaks in the same season. Abundance dropped abruptly during the coldest and driest months, from May to August. Frequently none of these species were recorded during June and July. This phenological pattern is similar to other Chrysomelidae living in subtropical areas of Brazil. Temperature and rainfall appear to be the major factors influencing the fluctuation of these three species.

## Introduction

Phenology can be considered a temporal dimension of natural history, and because both include timing of growth, reproduction and senescence, they are sometimes used as synonyms (Forrest and Miller-Rushing 2010). However, phenology does not include non-temporal aspects of life history, which in turn can affect phenology. The same authors point out that the proximate causes of phenological events are a combination of an organism’s genes and several external environmental factors, such as temperature, precipitation and photoperiod.

The role of abiotic variables in species phenology increases concern in how climatic change will affect species’ temporal and spatial distributions. Efforts are being made to predict biotic responses in relation to abiotic changes (see references in Forrest and Miller-Rushing 2010, Cleland et al. 2012). Several studies were recently started on mountains, since differing altitudes can simulate a gradient of environmental conditions similar to increasing latitude but within a small geographical range (Hodkinson 2005), thereby facilitating ecological research. Distribution and population fluctuations of phytophagous insects on mountains depend on a sum of factors such as thermal requirements for growth, temperature tolerance, dispersal ability, host plant quality and distribution, phenological synchrony with host plants, and interactions with competitors, parasites and predators (Alonso 1999, Obermaier and Zwolfer 1999, Lazzari and Lazzarotto 2005, references in Hodkinson 2005, Merrill et al. 2008).

Interestingly, previous studies on Chrysomelidae in Brazil (e.g. Nogueira-de-Sá et al. 2004, Flinte et al. 2009, 2010, 2011) showed that tropical species on mountains exhibit population fluctuations similar to species in subtropical areas, i.e. these beetles did not occur throughout the year as their tropical lowland neighbors, but rather had a restricted occurrence and disappeared during a period of the year. Below we describe aspects of the life history and phenology of three leaf beetle species in a tropical montane forest.

## Methods

Surveys were undertaken along the main road of the Serra dos Órgãos National Park (22°26'56"S and 42°59'5"W) in the county of Teresópolis, at approximately 1000 m altitude, characterized by montane rain forest. The Park lies in a mountainous area of the State of Rio de Janeiro, southeast Brazil, with elevations extending from 80 to 2263 m a.s.l. The climate in the region is tropical mesothermic (Köppen 1936), with a short dry season, mild summers and lower temperature due to the altitude. During the study, June to August were the coldest (mean temperature of 15.2 °C) and driest (mean of 81.1 mm monthly rainfall) months, while December to February were the warmest (mean temperature of 20.9 °C) and wettest (mean of 350.2 mm monthly rainfall) months (data from the National Institute of Meteorology at 980 m altitude, from 2008 to 2011).

Our study focuses on three abundant chrysomelid species previously observed by the author in the area, two cassidines and one chrysomeline. Study periods varied by species, but generally corresponded to the period from November 2008 to June 2011. Host plants were marked and thoroughly inspected for insects in periodic surveys, but it was not uncommon for plants to be accidentally cut down or to disappear during the study, which can explain some differences in host numbers between consecutive surveys. Host plant numbers and survey periods are presented below for each study species.

Coptocycla (Podostraba) arcuata (Swederus, 1787) (Cassidinae: Cassidini) feeds on the small shrub, *Cordia polycephala* (Boraginaceae) (Flinte et al. 2008), and was observed once or twice per month from November 2009 to June 2011. This species also feeds on *Cordia urticifolia* in the area (Flinte et al. 2008), but in a much lower frequency (Flinte, pers. obs.). Twenty plants of *Cordia polycephala* were surveyed from November 2009 to July 2010, and 30 plants from August 2010 to June 2011, an overall total of 20 months and 33 surveys.

*Omaspides* (*s. str.*) *trichroa* (Boheman, 1854) (Cassidinae: Stolaini) feeds on the vine *Ipomoea philomega* (Convolvulaceae) (Flinte et al. 2008) and was surveyed one to four times per month from November 2008 to March 2011, a total of 29 months and 69 surveys. Because leaves almost completely disappeared during some months of the year, the number of plants inspected varied between five and 24.

*Platyphora axillaris* Germar, 1824 (Chrysomelinae) feeds on the shrub *Solanum scuticum* (Solanaceae) and was studied from February 2009 to June 2011 (between one and four surveys per month). A total of 29 months and 78 surveys were conducted for this species. The number of plants surveyed varied from 14 to 46. There are no published accounts of this species.

During inspection, adults and larvae of all species, and eggs of *Omaspides trichroa*, were counted and observations were made regarding life history and behavior traits. Beetles were on occasions brought to the laboratory and reared in plastic containers with host plant leaves to complement field observations and to obtain parasitoids. Parasitized egg masses of *Omaspides trichroa* found in field were brought to the lab to obtain parasitism rates within clutches. The total number of records of adults and larvae on upper and lower sides of the leaves was also registered. Although it is possible that the same individual was recorded more than once, adults and larvae of the studied species are mobile, so their location could vary from one survey to another. Beetle, plant and parasitoid specimens are deposited in the collection of the Laboratório de Ecologia de Insetos, Universidade Federal do Rio de Janeiro, Brazil.

Due to the different numbers of host plants inspected on each survey we calculated the density of insects per plant for each beetle species to describe patterns of phenology. In addition to adults and larvae, densities were also calculated for egg masses and larval aggregations of *Omaspides trichroa*, and for young larvae of *Coptocycla arcuata*, as eggs are difficult to find in field. Densities were calculated separately for each survey, multiplied by 100 (to avoid decimals) and finally the mean density was determined for each month. Thus, a monthly mean density of 200, for example, indicates that for that stage, on average, 200 individuals were found per 100 plants on a given month, or two individuals per plant. Plant phenology was evaluated simply by the presence or absence of new leaf shoots for each individual inspected on each survey. The percentage of host plants with new shoots was calculated for each survey, dividing the number of plants with new shoots by the number of plants inspected and multiplying the result by 100. The mean percentage was then calculated for each month. The monthly mean density of beetles was correlated (Pearson correlation) with plant phenology and climate variables (temperature and rainfall). Lagged correlations of one, two and three months were made as well. The percentage of plants occupied by beetles (adult or larva) was also calculated to evaluate the intensity of attacks on plants and the spatial distribution of beetles on their host plants. Here, for each survey, the number of attacked plants was divided by the number of surveyed plants and multiplied by 100 to be expressed as percentage; then the mean and maximum percentages (considering all surveys) were established.

## Results

### Biological and behavioral traits

**Figure 1.**
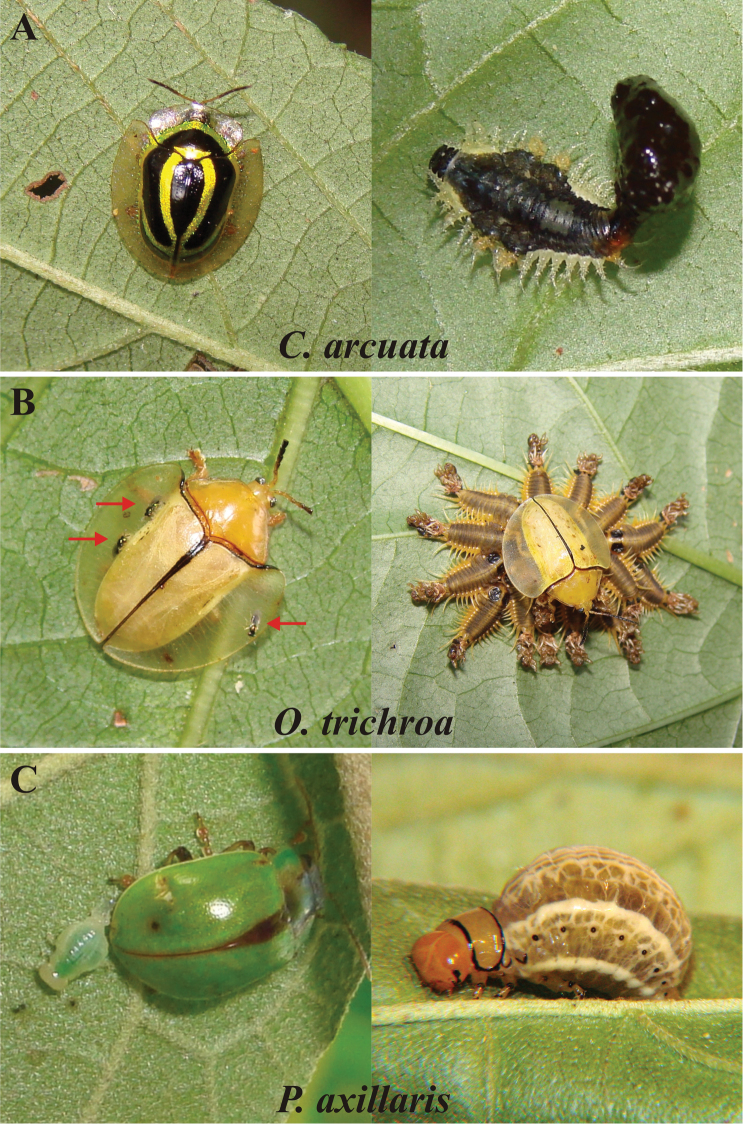
Adults and larvae of the study species: *Coptocycla arcuata* (**A**), *Omaspides trichroa* (**B**) and *Platyphora axillaris* (**C**), in a montane rain forest in southeast Brazil. Arrows in B show phoretic wasps.

*Coptocycla arcuata* deposits single, flattened, membranous eggs, and *Omaspides trichroa* deposits a mass of hard elliptical eggs which are guarded by the mother; both species lay eggs on the underside of leaves (n = 6 for *Coptocycla arcuata* and n = 167 records for *Omaspides trichroa*). The solitary larvae of *Coptocycla arcuata* carry an exuvio-fecal shield, resembling an elliptical blob of wet feces (Fig. 1A). The larvae of *Omaspides trichroa* occur in large (59.6 + 16.1 (n = 113)) maternally-guarded aggregations. Individual larvae bear a reduced shield composed more of exuvia than feces (Fig. 1B). Larvae of both species exhibited repeated and rapid flexing movements of shields when disturbed. Adults of *Coptocycla arcuata* tended to fly when approached. *Platyphora axillaris* is larviparous and its greenish larvae are deposited singularly and remain solitary throughout their development, later becoming brownish in colour (Fig. 1C). No maternal care was observed for this species. When manipulated, larvae regurgitate, as do adults, which additionally feign death as a defense. Larvae of all three species and adults of *Omaspides trichroa* were mainly found on the underside of leaves, while adults of *Coptocycla arcuata* and *Platyphora axillaris* preferred the upper side (Table 1). Field observations showed that *Coptocycla arcuata* pupates on the underside of leaves (n = 22 records) and *Omaspides trichroa* on stems (n = 17 records of aggregations). Pupae of *Platyphora axillaris* were only observed in the lab, buried in the soil placed at the bottom of the rearing container.

**Table 1.** Percentage and total number of records of adults and larvae (larval aggregations for *Omaspides trichroa*) on the upper and lower side of host plant leaves.

Species	Stage	Upper side (%)	Lower side (%)	N
*Coptocycla arcuata*	Adults	61.5	38.5	422
	Larvae	1.5	98.5	67
*Omaspides trichroa*	Adults	2.9	97.1	888
	Larval aggregations	0.0	100.0	190
*Platyphora axillaris*	Adults	76.2	23.8	632
	Larvae	8.3	91.7	223

The phoretic wasp *Emersonella pubipennis* Hansson, 2002 (Hymenoptera: Eulophidae) (Fig. 1B) oviposited on freshly laid eggs of *Omaspides trichroa*. Egg parasitism within clutches was 97.9% (ranging from 95 to 100%; n = 8 egg masses), but the percentage of parasitized clutches in the population is unknown. Even when parasitized, *Omaspides trichroa* mothers continued to care for their eggs. For this species we also obtained *Brachymeria* sp. (Hymenoptera: Chalcididae) parasitoids from pupae, and a tachinid fly species from prepupa. On one occasion, a Vespidae wasp was observed attacking a group of larvae without a caring mother (Khattar, pers. comm.). It approached the group, grasped a larva, turning it over, and flew away, returning soon after to grasp another one. When the same wasp moved towards a nearby group of larvae with attending mother, the mother made rapid movements in its direction, driving the wasp away. An unidentified species of tachinid fly emerged from a *Platyphora axillaris* larva brought from the field.

### Population fluctuations

**Figure 2.**
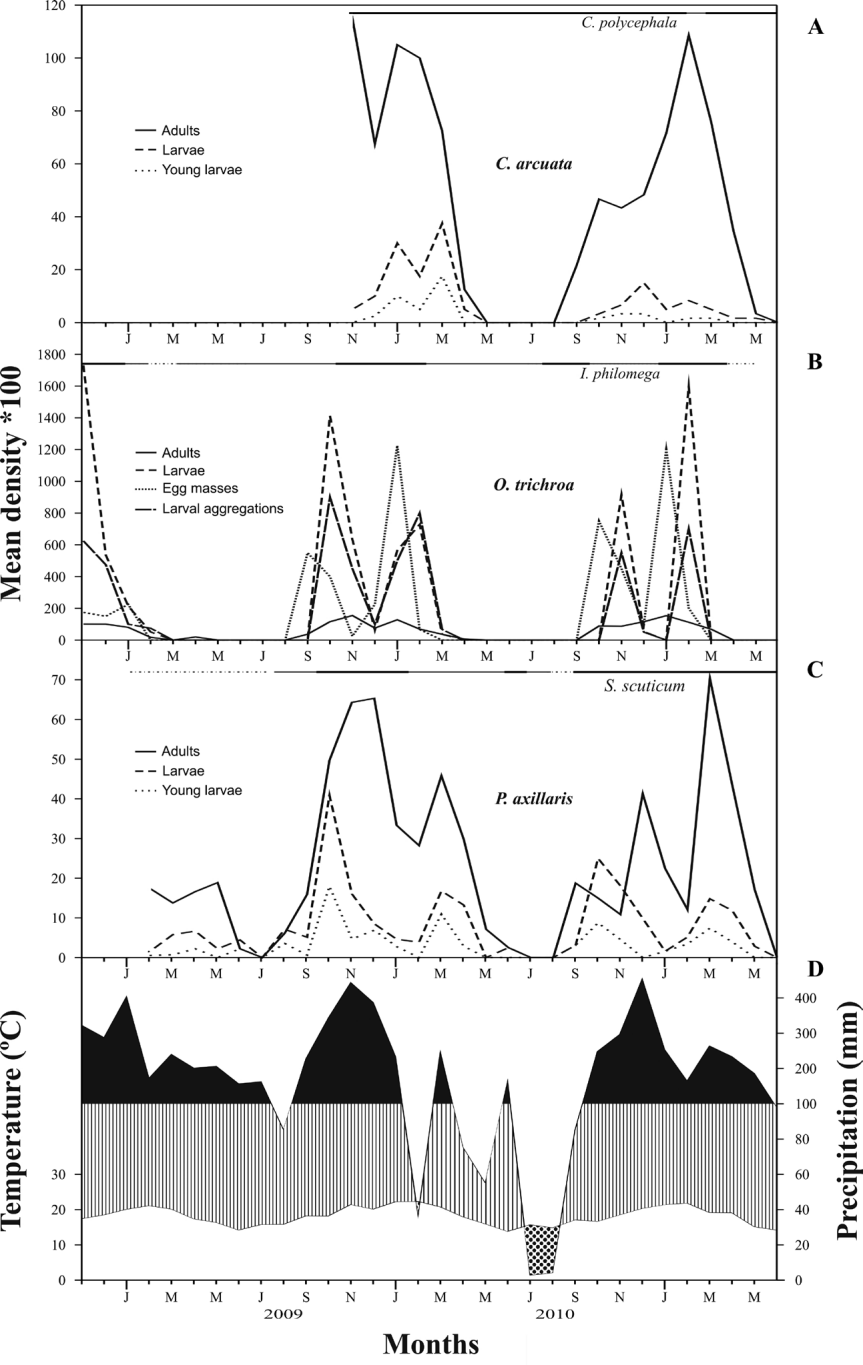
Population phenology of *Coptocycla arcuata* (**A**), *Omaspides trichroa* (**B**) and *Platyphora axillaris* (**C**) in a montane forest at 1000 m altitude between November 2008 and June 2011. A climatic diagram (data obtained from a meteorological station in the same site) is given for the same period as surveys (**D**). Dotted area = dry period; striped area = humid period; black area = super-humid period. The line above species fluctuations represents the percentage of host plant with new leaf shoots, the dotted line being < 25% of plants in this phase; fine line between 25% and 75%; thick line > 75%.

Densities of the three chrysomelid species varied similarly throughout the year, with higher numbers from October to March (spring and summer), and lower numbers or even absence of beetles from June to August (Fig. 2). The period of absence varied between species, being shorter in *Platyphora axillaris* (one or two months) and larger in *Omaspides trichroa* (five months). Fluctuations in densities (per 100 plants) of different stages also followed similar trajectories for the three species, as follows.

*Omaspides trichroa* was studied during two whole reproductive seasons, 2009/2010 and 2010/2011. Adults and egg masses started to be found in September 2009 in the first season and in October 2010 in the second season, and larvae always appeared one month later. Densities of egg masses and larvae peaked right away, then decreased abruptly in November (eggs) and December 2009 (larvae), increasing again and peaking once more in January (eggs) and February 2010 (larvae). Exactly the same pattern was observed in the following season, with exception that densities of eggs and larvae decreased together in December 2010. Density of adults also showed this bimodal pattern of occurrence in two consecutive seasons. Densities of all stages then decreased rapidly and disappeared completely from May until the following season. Studies on *Coptocycla arcuata* started in the beginning of the reproductive season 2009/2010 and extended until the end of the season 2010/2011. Adults were first found in September, and larvae one month later. Density of young larvae (as an approximation of the egg stage) and total larval density varied similarly, peaking in the first season in January 2010 and in the second season in December 2010, decreasing and reaching another peak in March 2010 and February 2011. Adults of this species also showed two peaks of occurrence. *Platyphora axillaris* was studied also during seasons 2009/2010 and 2010/2011. Densities of adults peaked at least twice in each reproductive season, once or twice in the end of the year and then again in March, and their larvae earlier in October and a second time in March, during two consecutive seasons. Overall, densities of all stages showed two peaks in each reproductive season for the three species, suggesting the existence of two generations per year, i.e. bivoltine reproduction. Numbers of all species decreased in April and some completely disappeared in subsequent months, increasing again in September (Fig. 2).

The seasonal fluctuation of beetle densities correlated with variations of temperature and rainfall throughout the year, with high numbers coinciding with super-humid periods (precipitation above 100 mm per month) and warmer months in the study area (Fig. 2). Significant correlations were obtained in most cases between beetle densities, both adult and larval, and temperature and precipitation values (Table 2). On the other hand, plant phenology, more specifically the presence of new leaf shoots, was only positively related to density of *Omaspides trichroa* adults, but not for larval aggregations. The majority of host plants of the two other species seem to be adding new leaves throughout the year (Fig. 2), although neither the intensity of this production per plant nor the nutritional quality of leaves were measured. Lagged correlations of one, two and three months did not show more significant results.

**Table 2.** Correlations (Pearson) between monthly mean density of adults and larvae of *Coptocycla arcuata*, *Omaspides trichroa* and *Platyphora axillaris* and monthly mean temperature, total precipitation per month and monthly mean percentage of plants with new shoots. For *Omaspides trichroa* densities of larval aggregations are given. Number of months used are given by n.

Species	Stage	Temperature (°C)	Precipitation (mm)	Plants with new shoots
*Coptocycla arcuata*	adults	r = 0.922*** (n=20)	r = 0.493* (n=20)	r = -0.191 (n=20)
	larvae	r = 0.655** (n=20)	r = 0.267 (n=20)	r = 0.014 (n=20)
*Omaspides trichroa*	adults	r = 0.665*** (n=29)	r = 0.702*** (n=29)	r = 0.410* (n=29)
	aggregations	r = 0.422* (n=29)	r = 0.260 (n=29)	r = 0.280 (n=29)
*Platyphora axillaris*	adults	r = 0.608*** (n=29)	r = 0.728*** (n=29)	r = 0.319 (n=18)
	larvae	r = 0.218 (n=29)	r = 0.558** (n=29)	r = 0.336 (n=18)

* *p* < 0.05; ** *p* < 0.01; *** *p* < 0.001

Although fluctuation patterns were similar among species, their numbers could differ by an order of magnitude, especially for *Omaspides trichroa*, in which larval density reached 17 times that of the adults (for larval aggregations the number was six times higher than adults). For the other two species, adults were generally more abundant than larvae (Fig. 2). Maximum density was 2.7 adults per plant for *Omaspides trichroa*, 1.8 for *Coptocycla arcuata* and 0.9 for *Platyphora axillaris*. Larval maximum density was 37.0 individuals per plant for *Omaspides trichroa*, but considering larval aggregations, maximum density dropped to 0.7 per plant, similar to 0.6 found for the larvae of the other two species. The mean percentage of plants occupied by any beetle, adult or larva, was 31% for *Omaspides trichroa*, 30.9% for *Coptocycla arcuata* and 16.1% for *Platyphora axillaris*, varying from a minimum of zero for all three species to a maximum of 70%, 80% and 38.9%, respectively, of attacked plants per survey.

## Discussion

This study describes aspects of the natural history and phenology for three Chrysomelidae species occurring at 1000 m altitude in SE Brazil. Our results document that, despite differences in life history traits occurring among these three taxonomically distinct species, all present a similar two-peak pattern of reproduction during the warmest and wettest months of the year. Below our findings are discussed in the context of previous reports on Neotropical Chrysomelidae.

Earlier studies documented that *Omaspides trichroa* feeds on only a single plant species at the study area, whereas *Coptocycla arcuata* feeds on two related plants (Flinte et al. 2008), which was confirmed in this work. Field observations also confirmed the findings of these authors that *Coptocycla arcuata* and *Omaspides trichroa* are oviparous and construct an exuvio-fecal shield as larvae (Fig. 1A, B). However, the observations that *Platyphora axillaris* is larviparous (Fig. 1C) and feeds on *Solanum scuticum*, are unique and reported here for the first time. *Platyphora* is a neotropical genus (Daccordi 1996), containing species closely associated with Solanaceae host plants (Jolivet and Hawkeswood 1995), with viviparous habits (Vasconcellos-Neto and Jolivet 1994, Bernardi and Scivittaro 1991, Schroder et al. 1994, Olckers 2000, Windsor et al. 2013) and some species showing maternal care (Windsor et al. 2013). Together with egg covering, female site selection and maternal care, viviparity is likely a mean of defending the vulnerable egg stage (Hilker 1994).

The preferences of adults of *Coptocycla arcuata* and *Platyphora axillaris* for the upper side of leaves (Table 1) can be related to possible defenses of aposematic and cryptic coloration, respectively, but experiments are necessary to test this hypothesis. Additionally, many species of *Platyphora* are known to have chemical defenses, secreting secondary plant metabolites through elytral and pronotal exocrine glands (Pasteels et al. 2001). On the other hand, adults of *Omaspides trichroa*, mainly represented by mothers, were found more often on the underside of leaves, where they can protect their young inconspicuously. Larvae of all species were recorded frequently on the underside of leaves, where it is harder for flying visual predators to detect them. Nogueira-de-Sá and Vasconcellos-Neto (2003) obtained similar results for young larvae of three other Cassidinae species and suggest that their position is explained by female site selection, in an effort to protect immature stages from visually oriented natural enemies and unfavorable abiotic conditions. Indeed, it has recently been shown that maternal oviposition choices (site/leaf) significantly impact the effectiveness of larval tortoise beetle defenses and survival (Vencl et al. 2013). The fleshy larvae of *Platyphora axillaris* seem especially vulnerable compared to those of Cassidinae, which have a dorsal shield (a common defense in the subfamily – Olmstead 1996) and/or maternal care (a defense limited to Chrysomelinae and Cassidinae – Windsor and Choe 1994). However, they do regurgitate when disturbed, a potentially defensive behavior amongst many chrysomelids (Pasteels et al. 1988). Nonetheless parasitoids were capable of circumventing these defenses as were found attacking *Omaspides trichroa* and *Platyphora axillaris*. Cassidinae and Chrysomelinae are frequently parasitized by Hymenoptera and Tachinidae (Diptera) (Cox 1994, 1996). According to Cuignet et al. (2008), more than half of the tortoise beetle species of Panama have egg parasitoids of the Eulophidae family, mostly *Emersonella* spp., while larvae and pupae parasitoids are mainly of *Brachymeria* and *Conura* (Chalcidae) species. Interestingly, an egg parasitoid, *Emersonella pubipennis*, was foretic on female *Omaspides trichroa* adults, and a *Brachymeria* species attacked pupae, similar to the pattern found in Panama (Cuignet et al. 2008). Besides these parasitoids, a vespid wasp was observed predating larvae of *Omaspides trichroa* in a group without attending mother. Although most accounts of predation on cassidines are by piercing/sucking insects, mandibulate predators have been found attacking cassidines that lack or have reduced shields (Cox, 1996), as is the case of *Omaspides trichroa*.

Clearly, the three Chrysomelidae species have very different life histories and a distinct array of defenses. *Platyphora axillaris* is larviparous with no maternal care, while *Omaspides trichroa* is oviparous, with females caring for their young and larvae carrying an exuvio-fecal shield on their dorsum; both species are monophagous at the study area. *Coptocycla arcuata*, on the other hand, is oligophagous, feeding on two plant species, but oviparous without maternal care and with larvae also carrying a shield. Despite these significant differences between the three species, they showed very similar population phenologies (Fig. 2), which may suggest that external factors are mostly influencing their fluctuations. Adult densities for all species were significantly related to temperature and precipitation, and in the case of *Omaspides trichroa* also to leaf flushing, while larval densities varied: in *Coptocycla arcuata* and *Omaspides trichroa* they were related to temperature, and in *Platyphora axillaris* to precipitation. Thus, climate seems to affect the species fluctuations directly and, at least for *Omaspides trichroa*, also indirectly via host plant. Indeed, climate and/or resources are frequently used to explain insect population fluctuations (e.g. Wolda 1978, Demster and Pollard 1981, Medeiros and Vasconcellos-Neto 1994, Monteiro and Macedo 2000, Nogueira-de-Sá and Vasconcellos-Neto 2003, Nogueira-de-Sá et al. 2004).

Our findings corroborate other studies on Chrysomelidae in the same area (Flinte et al. 2009, 2010, 2011), where species reproduce during the hot rainy months and practically disappear when temperatures and rainfall drop, probably undergoing diapause as adults. Although taking place in a tropical latitude, the study was conducted at ca. 1000 m altitude, which approximates subtropical conditions. In their review on population phenologies of tortoise beetles in Brazil, Nogueira-de-Sá et al. (2004) discuss that climate tends to be more important for cassidines in subtropical areas, where species usually have a well defined reproductive season and overwinter in diapause. This is clearly supported by Medeiros and Vasconcellos-Neto (1994) studying five chrysomeline species on Solanaceae in South Brazil. One of these species, *Platyphora anastomozans*, showed two peaks in its reproductive season, in December and in March, exactly like the *Platyphora* species in this study. The existence of two well defined abundance peaks in the same reproductive season observed in our study indicates two generations per year occurring in the favorable season (spring and summer), explained as follows. After overwintering, adults immediately start laying eggs (in the case of *Coptocycla arcuata* and *Omaspides trichroa*) or larvae (for *Platyphora axillaris*), generating the first peak in abundance. The emerging adults lead to the second peak of abundance, and they reproduce in the same season, resulting in a second generation of immatures. By the end of the reproductive season, adults overwinter during the driest and coldest months (Fig. 2). The importance of climate in species phenology is proven by the strong correlations found between the different stages and variables of temperature and precipitation (Table 2). It is interesting to notice that there are also two distinct peaks of precipitation in each of the seasons 2009/2010 and 2010/2011, in November and March, and in December and March, respectively.

Variation in the order of magnitude of species numbers, especially the much larger density of *Omaspides trichroa* larvae, can possibly be explained by the behavior of maternal care, which increases immature survivorship (Windsor and Choe 1994, Chaboo et al. 2014). Thus, maximum density of *Omaspides trichroa* larvae per plant was much higher than for the other species. However, considering *Omaspides trichroa* larval aggregations, their maximum density on plants was similar to larvae of the other species, ca. 0.6 per plant. Adult maximum density ranged from 0.9 individuals per pant in *Platyphora axillaris* to 2.7 in *Omaspides trichroa*, i.e. more or less 1 individual per plant on periods of high abundance. Considering that, on average, no more than 31% of each plant species was occupied by any specimen of the studied species, these densities on plants indicate that beetles are not evenly distributed on their host plants. Nevertheless, attack on plants varied greatly along the year and were more intense on the months of higher densities, reaching 80% of plants attacked by *Omaspides trichroa* in one of the surveys.

Despite considerable differences in life history traits and systematic position among the three chrysomelid species, our data suggest that they are bivoltine, disappearing during the unfavorable period of lower temperatures, a pattern similar to species in subtropical regions and other species already studied in the same area. The climatic variables of temperature and precipitation seem to be important drivers for species phenologies. In light of the undergoing climatic changes, studies on insects and their host plants on mountains can provide an important tool for studying the responses of species to changing environmental conditions, predicting possible future scenarios and collaborating with species conservation efforts.
